# Aberrant baseline brain activity and disrupted functional connectivity in patients with vascular cognitive impairment due to cerebral small vessel disease

**DOI:** 10.3389/fneur.2024.1421283

**Published:** 2024-07-03

**Authors:** Ronghua Mu, Peng Yang, Xiaoyan Qin, Wei Zheng, Xin Li, Bingqin Huang, Xiqi Zhu

**Affiliations:** ^1^Department of Radiology, Nanxishan Hospital of Guangxi Zhuang Autonomous Region, Guilin, China; ^2^Graduate School, Guilin Medical University, Guilin, China; ^3^Department of Radiology, Affiliated Hospital of Youjiang Medical University for Nationalities, Baise, China; ^4^Life Science and Clinical Medicine Research Center, Affiliated Hospital of Youjiang Medical University for Nationalities, Baise, China

**Keywords:** cerebral small vessel disease, vascular cognitive impairment, resting-state functional magnetic resonance imaging, fractional amplitude of low-frequency fluctuation, functional connectivity

## Abstract

**Objective:**

This study aims to examine the alterations in aberrant brain activity and network connectivity between individuals with mild and major vascular cognitive impairment (VCI).

**Materials and methods:**

A total of 114 patients with cerebral small vessel disease (CSVD) were included in this study, comprising 61 individuals with mild VCI (mean age, 55.7 ± 6.9 years; male, 42.6%) and 53 cases with major VCI (mean age, 57.6 ± 5.5 years; male, 58.5%). Additionally, 53 age-, gender-, and education-matched healthy subjects were recruited as normal controls (NC) (mean age, 54.9 ± 7.9 years; male, 52.9%). All participants underwent neuropsychological assessments and magnetic resonance imaging scans. One-way analysis of variance was used to compare fractional amplitude of low-frequency fluctuation (fALFF) values among the three groups. Two-sample t-tests were conducted to assess functional connectivity matrices between different groups for each connection. Moreover, mediation analyses were performed to explore the mediating effect of aberrant brain activity on the relationship between cognitive impairment and CSVD total burden.

**Results:**

VCI patients exhibited aberrant brain activity in regions such as the right thalamus (THA_R), right cuneus (CUN_R), left postcentral gyrus (PoCG_L), right postcentral gyrus (PoCG_R), right median cingulate, paracingulate gyri (PCG_R), and left precuneus (PCUN_L). Reduced positive functional connectivity was predominantly observed among nodes including PCUN_L, CUN_R, PoCG_L, PoCG_R, right posterior cingulate (PCG_R), and left occipital gyrus (IOG_L) in VCI patients. The aberrant baseline brain activity and disrupted brain network were more pronounced with worsening cognitive function. Increased fALFF values in THA_R, CUN_R, and PoCG_L mediated cognitive impairment in CSVD patients.

**Conclusion:**

Abnormal brain activities in THA_R, CUN_R, and PoCG_L, along with their associated abnormal functional connections, play a significant role in VCI. The study revealed a progressive increase in aberrant brain activity and network connectivity with advancing stages of VCI.

## Introduction

CSVD (cerebral small vessel disease) is a condition characterized by impaired microcirculation in the brain and is a leading cause of vascular cognitive impairment (VCI) (1). It is prevalent among middle-aged individuals and its prevalence increases with age (2). As life expectancy rises in aging societies, VCI poses a significant threat to the elderly population in China, leading to a growing economic burden on families and society (3). The heterogeneous nature of CSVD has made it challenging for researchers to identify reliable biomarkers that can elucidate the common mechanisms underlying VCI (4). Imaging markers obtained through magnetic resonance imaging (MRI) play a crucial role in understanding the pathophysiology and clinical progression of CSVD. These markers include lacunar infarction, cerebral microbleed, white matter hyperintensity, and enlarged perivascular spaces (5). While these imaging markers are valuable in reflecting cognitive impairment in CSVD patients, the presence of multiple pathological changes in the aging brain and an increased total load of CSVD imaging markers can weaken the predictive capacity of individual markers for cognitive impairment over time (6). Developing and validating a non-invasive protocol for predicting VCI is of utmost clinical importance for enabling early intervention and treatment of VCI. Such a protocol could help in the identification of at-risk individuals and potentially aid in reducing the burden of VCI on individuals and society.

Resting state functional MRI (rs-fMRI) has emerged as a valuable tool for studying brain activity due to its ability to detect brain oxygen levels with high spatial resolution. This sensitivity makes rs-fMRI particularly useful for investigating the neural basis of VCI. One important metric used in rs-fMRI studies is the amplitude of low-frequency fluctuations (ALFF), which reflects the total power of the blood oxygenation level dependent (BOLD) time course and provides insight into spontaneous brain activities (7). Fractional ALFF (fALFF) is another metric that can help reduce interference from physiological signals, thereby enhancing the detection of spontaneous neural activity (8). As a non-invasive imaging technique, fALFF allows for the detection of intrinsic brain activities associated with VCI. Previous studies have identified aberrant fALFF in various brain regions such as the precentral gyrus, superior parietal gyrus, parahippocampal gyrus, and inferior temporal gyrus in VCI patients (9). The functional connectivity (FC) analysis focuses on the coherence of activity within and between brain regions, measuring the strength of connections between regions with shared functional properties (10). Abnormal FC patterns suggest disruptions in specific brain networks, indicating underlying brain dysfunction (11). Study has reported abnormal FC in regions such as the inferior frontal gyrus, middle frontal gyrus, precentral gyrus, and postcentral/superior parietal lobule in VCI patients (12), emphasizing the impact of disrupted brain network connections on cognitive impairment. As cognitive impairment worsens, there is evidence to suggest that brain activity and network connectivity disruptions may also worsen, with an increasing number of decreased connections between brain regions (13). The progression of cognitive impairment is associated with disruptions in various brain areas, including thalamo-cortical, dorsal attention, visual, and sensorimotor regions (14). In future VCI research, it will be important to further investigate how abnormal brain activity and network disruptions evolve as cognitive impairment worsens, which additional brain regions are affected, and how these abnormalities contribute to the clinical manifestations of CSVD and cognitive impairment.

The aim of this study is to investigate the alterations in aberrant brain activity and network connectivity in individuals with mild and major VCI resulting from CSVD. The study hypothesis posits that the changes in abnormal brain activity will intensify progressively as cognitive impairment worsens. Additionally, it is anticipated that as mild VCI advances to major VCI, there will be increased or varied involvement of brain regions, which could serve as critical indicators of cognitive decline. Understanding the differences and progression of abnormal brain activity across varying degrees of cognitive impairment can provide valuable insights into the pathological processes driving cognitive deterioration in individuals with CSVD. By exploring these disparities and changes in abnormal brain activity, this study aims to shed light on the underlying mechanisms contributing to the development of cognitive impairment in CSVD patients.

## Subjects and methods

### Subjects

The study was conducted at Nanxishan Hospital of Guangxi Zhuang Autonomous Region from May 2020 to December 2023 and was approved by the Ethics Committee of the hospital (2020NXSYEC-006). Written informed consent was obtained from all participants or their legal guardians. This study is a prospective cross-sectional study, including a total of 114 patients with CSVD, among whom 61 were classified as having mild VCI and 53 were classified as having major VCI. Additionally, 53 healthy subjects matched in terms of age (*p* = 0.119), gender (*p* = 0.225), and education (*p* = 0.075) were recruited as normal controls (NC). Patients were categorized as having CSVD-related VCI based on imaging criteria batteries of cognitive assessment scales, including the presence of lacunar infarctions and/or cerebral microbleeds, moderate to severe enlarged perivascular spaces >20, and deep white matter hyperintensity ≥ Fazekas 2 or periventricular white matter hyperintensity > Fazekas 3 (15). Each item above is scored one point, and the overall burden of CSVD is evaluated using the total score of CSVD.

Exclusion criteria for the study encompassed individuals with a history of psychiatric or neurological disorders, alcohol or substance abuse, craniocerebral operations, liver, kidney, or other vital organ damage, visual or hearing impairments, and systemic illnesses that may impact cognition.

### Collection of demographic and clinical data

Trained medical staff collected demographic, epidemiological and clinical data through face-to-face interviews, clinical examinations, and neuropsychological tests. The data were collected using structured questionnaires, which included demographic features (age, sex, education, and occupation), lifestyle habits (such as smoking and alcohol consumption), and vascular risk factors (hypertension, diabetes mellitus, coronary heart disease, lacunar infarction, carotid atherosclerosis, etc.).

### Neuropsychological assessment

Multiple cognitive domains, such as executive function, memory, attention, visuospatial skills, and orientation, were evaluated using the Beijing version of the Montreal Cognitive Assessment (MoCA), scoring up to 30 points. The severity of VCI was determined using the Activities of Daily Living instrument, assessing independence in daily activities. In accordance with the guidelines of Vascular Impairment of Cognition Classification Consensus, mild VCI was identified in patients where daily living independence remained unaffected or mildly affected, while major VCI was diagnosed in patients with significant impairment in daily living independence (5).

### MRI protocols of data acquisition

The MRI examinations were conducted using a 3.0 T MRI system (Ingenia 3.0CX; Philips Healthcare, Best, the Netherlands) with 32-channel head coils. Various three-dimensional (3D) MR parameters were employed in this study, including 3D T1, 3D T2, and 3D FLAIR sequences. The specific scan sequences utilized were as follows: The 3D T1-weighted imaging employed a fast field echo sequence with a 6.4 ms repetition time (TR) and a 3.0 ms echo time (TE). The field of view (FOV) was 240 mm × 240 mm × 180 mm, reconstruction voxel size was 1.1 × 1.1 × 1.1, the reconstruction matrix was 512 × 512, and the slice thickness was 1.1 mm. For the 3D T2 spin echo sequence, the TR was 2,500 ms, TE was 232 ms, FOV was 250 × 250 × 180 mm, reconstruction voxel size was 1.1 × 1.1 × 1.1, the reconstruction matrix was 512 × 512, and the slice thickness was 1.1 mm. The 3D FLAIR sequence had a TR of 4,800 ms, TE of 244 ms, FOV of 240 mm × 240 mm × 173 mm, reconstruction voxel size of 1.1 × 1.1 × 1.1, reconstruction matrix of 384 × 384, and slice thickness of 1.2 mm. Additionally, diffusion-weighted imaging (DWI) was performed with a TR of 2,462 ms, TE of 63 ms, FOV of 230 mm × 230 mm × 143 mm, reconstruction matrix of 192 × 192, slice thickness of 5 mm, reconstruction voxel size of 1.5 × 2.2 × 5, and 24 axial slices. For susceptibility-weighted imaging (SWI), the TR was 29 ms, TE was 7.2 ms, FOV was 230 mm × 189 mm × 130 mm, reconstruction matrix was 768 × 768, slice thickness was 2 mm, reconstruction voxel size was 0.6 × 0.6 × 2, and there were 130 axial slices. Resting-state fMRI parameters were TR = 2000 ms, TE = 30 ms, flip angle = 90°, FOV = 240 × 240 × 144 mm, reconstruction voxel size = 2.5 × 2.5 × 2.5, reconstruction matrix = 96, slice thickness = 3.6 mm, no spacing between layers, 40 scan layers, and NSA = 1, the acquisition of this sequence lasted approximately 8 min, encompassing 240 functional volumes per subject. Each participant underwent the aforementioned MRI scan protocol, which lasted approximately 30 min.

### Quality control

Participants were instructed to keep their eyes open, remain awake, breathe regularly, and refrain from engaging in specific thoughts during the procedure. Following the scan, participants were queried about any instances of nodding off, which were duly noted. All images underwent manual scrutiny to assess data quality, with those exhibiting significant artifacts, irregularities, and/or poor quality excluded from further processing and analysis. A radiologist and technician jointly verified the aforementioned procedures.

### Structural MRI analysis

Gray matter, white matter, and cerebrospinal fluid (CSF) were automatically segmented from structural 3D T1 brain images using the CAT12 toolbox integrated into Statistical Parametric Mapping 12 (SPM 12). The total intracranial volume (TIV) was calculated by summing the volumes of gray matter, white matter, and CSF. The processing steps involved: (1) Segmentation of T1-weighted images into gray matter, white matter, and CSF using the unified segmentation module. (2) Generation of study-specific group templates using Diffeomorphic Anatomical Registration using Exponentiated Lie algebra (DARTEL) to achieve robust inter-subject registration. (3) Normalization of each participant’s brain to the MNI space with the group brain template as the reference image. The normalized images were modulated to maintain relative gray matter volumes post-spatial normalization. (4) The modulated gray matter images underwent smoothing with an 8 mm full width at half maximum (FWHM) Gaussian kernel filter. Subsequent verification of the output results was conducted by two expert radiologists.

### Resting-state fMRI analysis

The fMRI data preprocessing was conducted utilizing SPM12 and the Resting-state fMRI data analysis toolkit (REST plus).^1^ This process involved several steps, outlined as follows: (1) The initial 10 functional volumes were discarded to stabilize the fMRI signal and allow participants to acclimate. (2) Subsequently, slice-timing disparities between acquired images were corrected, and subjects exhibiting head movements exceeding 2.0 mm or rotations exceeding 2.0° were excluded to mitigate motion artifacts’ impact on the signal. (3) Normalization was executed using the DARTEL method with a new segmented T1 image, leading to voxel resampling at a size of 3 mm × 3 mm × 3 mm. (4) Nuisance covariate regression was performed to further diminish noise effects, incorporating white matter signals, CSF, global brain signals, and the standard six head motion parameters. (5) Detrending and filtering in the range of 0.01–0.08 Hz were implemented to eliminate low-frequency drifts and high-frequency physiological noise.

### Metrics calculation

#### fALFF

The ALFF represents the strength or intensity of low frequency oscillations and is defined as the total power within the low frequency range of 0.01–0.08 Hz. The fALFF is defined as the ratio of the power spectrum of the low-frequency range, 0.01–0.08 Hz, to that of the entire detectable frequency range (0–0.25 Hz), and it represents the relative contribution of specific low frequency oscillations to the entire frequency range. Because fALFF is assumed to be less sensitive to physiological noise, the functional time series of each voxel was transformed to the frequency domain using a fast Fourier transform algorithm, and the power spectrum was obtained. The power of a frequency is dependent on the square of its amplitude component, thus, the square root of each frequency in the power spectrum was calculated, and an averaged square root was taken across the range of 0.01–0.08 Hz for each voxel. Using the power spectrum of the low-frequency range, 0.01–0.08 Hz, and that of the whole frequency range, fALFF was derived through a ratio calculation. Z-score normalization was performed for statistical analysis to create zfALFF maps.

#### Seed-based functional connectivity analysis

We used a seed-voxel correlation approach to evaluate the functional connectivity of all participants based on gray matter of the brain. Brain regions that showed zfALFF alterations for patients were selected as regions of interest (ROIs), we calculated the resting-state functional connectivity between the average time series of the seed and the time series of each voxel in the gray matter remaining after removing the seed region. And computing Pearson’s correlation coefficients between them and the time course of all other voxels in the brain. Correlation coefficients were normalized to z values via Fisher’s z-transformation.

#### Network-based statistics

We identified group differences in sub-networks using the network-based statistic (NBS) approach implemented in the NBS (V1.2) toolbox^2^ to determine which nodes and connections are likely to have contributed to the differences in network density (16). Regions that showed zfALFF alterations in patients were selected as ROIs.

### Statistics

#### Demographic and clinical data

We conducted statistical analyses using Matlab and R software. Sample characteristics were compared among the NC, mild VCI, and major VCI groups using the Chi-squared test for categorical variables and analysis of variance for numerical variables.

#### Calculation of the fALFF value

Based on a gray matter template, one-way ANOVA analysis was applied using SPM 12 to investigate differences of whole-brain zfALFF values among NC, mild VCI, and major VCI groups, with age, education level, sex, and gray matter volume as covariates. Statistical analysis was performed voxel-wise and ROI-wise using SPM software. Multiple comparisons during the analyses were corrected using the Gaussian random field theory (significant for voxel levels at *p* < 0.001 and cluster at *p* < 0.05).

#### Calculation of the FC value

ROI-wise connectivity was measured by time course data from each ROI above and calculating the Pearson’s correlation coefficients between each ROI pair. The resulting correlation coefficients were then Fisher-transformed into ‘Z’ scores to increase normality. Two-sample t-test was used to compare connectivity matrices between pairs of groups at each connection. Connections with t-values higher than 3.1 were combined to create a sub-network, using the t-value as a descriptive threshold. The size of the sub-network was compared to the null distribution of sub-network sizes to obtain a *p*-value. To create the null distribution, the procedure was repeated 10,000 times with group labels randomly permuted. As stated previously, an adjusted significance level of ɑ = 0.05 was used. Age, education level, sex, and gray matter volume were used as covariates in the process mentioned above. We visualized statistically significant sub-networks using BrainNet Viewer (version 1.63).^3^

#### Mediation analysis

Furthermore, to investigate if the association between cognitive impairment across various subjects and the total load of CSVD was mediated by specific brain regions manifesting abnormal activity, we conducted mediation analyses with adjustments for age, education level, sex, and gray matter volume. We applied Bonferroni correction for multiple comparisons, and bilateral *p*-values below 0.05 were considered statistically significant, indicating significant differences.

## Results

### Demographic and clinical characteristics of the participants

One hundred and sixty-five subjects were included in the final analysis. Detailed demographic and clinical data are presented in Table 1. The MoCA scores were significantly lower in the major VCI group than those in the mild VCI group. There were no significant group differences among the three groups in age, sex, education, TIV, gray matter volume, and other Variables.

**Table 1:** Demographic and neuropsychological characteristics of the included subjects.

Characteristics	NC (51)	Mild VCI (61)	Major VCI (53)	F/χ^2^	*p* value
Demographics					
Age (y)	54.9 ± 7.9	55.7 ± 6.9	57.6 ± 5.5	2.160	0.119
Male (%)	27 (52.9)	26 (42.6)	31 (58.5)	2.979	0.225
Education (y)	11.9 ± 4.2	10.8 ± 4.3	9.9 ± 5.1	2.633	0.075
BMI (Kg/m2)	24.7 ± 2.7	24.8 ± 3.1	24.1 ± 2.8	0.772	0.464
Systolic pressure (mmHg)	136.8 ± 19.7	132.3 ± 18.9	136.1 ± 21.1	0.872	0.420
Diastolic pressure (mmHg)	85.5 ± 14.3	83.8 ± 12.3	84.8 ± 12.0	0.246	0.783
Smoking (%)	20 (39.2)	18 (29.5)	15 (28.3)	1.723	0.423
Drinking (%)	8 (15.7)	9 (14.8)	7 (13.2)	0.132	0.936
Diabetes mellitus (%)	4 (7.8)	3 (4.9)	7 (13.2)	2.549	0.280
Coronary heart disease (%)	3 (5.9)	2 (3.3)	2 (3.8)	0.506	0.777
Carotid atherosclerosis (%)	12 (23.5)	8 (13.1)	12 (22.6)	2.454	0.293
Snoring (%)	40 (78.4)	45 (73.8)	49 (73.6)	0.426	0.808
MoCA	27.7 ± 1.3	22.8 ± 2.3	14.8 ± 2.1	563.834	< 0.001^a^
GMV (ml)	574.817 ± 37.435	579.383 ± 45.387	572.856 ± 38.801	1.300	0.277
TIV (ml)	1484.343 ± 111.816	1474.579 ± 139.232	1442.223 ± 133.821	1.530	0.220
Neuroimaging markers [*n* (%)]					
LI	NA	8 (13.1)	9 (17.0)	0.334	0.563
WMH	NA	58 (95.1)	51 (96.2)	0.089	0.766
EPVS	NA	47 (77.0)	46 (86.8)	1729	0.181
CMBs	NA	13 (21.3)	19 (35.8)	2.968	0.085

NC, normal control; VCI, vascular cognitive impairment; SD, standard deviation; BMI, body mass index; GMV, gray matter volume; TIV, total intracranial volume; MoCA, Montreal Cognitive Assessment; LI, lacunar infarcts; WMH, white matter hyperintensities; EPVS, enlarged perivascular spaces; CMBs, cerebral microbleeds. In the post-hoc comparison, a represents NC < mild VCI < major VCI.

### Comparison of zfALFF between groups in voxel levels

**Figure 1:**
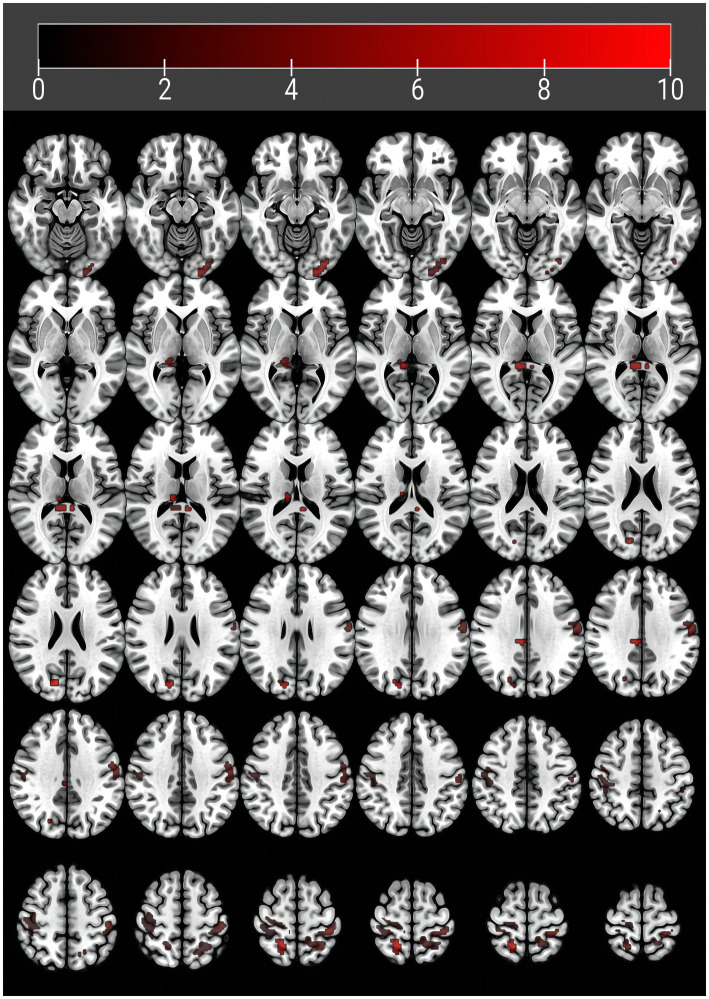
Brain regions with abnormal amplitude of low-frequency fluctuations in vascular cognitive impairment patients. Abnormal regions are detailed in Table 1.

In terms of the comparison of zfALFF, we found significant differences in the right thalamus (THA_R), right cuneus (CUN_R), left postcentral gyrus (PoCG_L), right postcentral gyrus (PoCG_R), right median cingulate and paracingulate gyri (DCG) and left precuneu (PCUN_L) regions across the three groups, in the post-hoc multiple comparisons, our results showed that zfALFF exhibited a tendency of major VCI < mild VCI < NC in these regions. However, we found the opposite tendency in the left inferior occipital gyrus (IOG_L), right posterior cingulate (PCG_R) and the right superior parietal gyrus (SPG_R), with NC < mild VCI < major VCI (Figure 1; Table 2).

**Table 2:** Differences in zfAlff values among groups.

Brain regions	Cluster size	Peak intensity	MNI coordinates of the peak voxel			F value	Post-hoc
			x	y	z		
IOG_L	32	40.4439	−24	−96	−12	15.071	A<B,C
PCG_R	30	43.2834	9	−39	9	105.004	A<B<C
THA_R	6	36.5988	9	−24	15	49.26	C<B<A
CUN_R	17	38.7423	15	−78	27	72.124	C<B<A
PoCG_L	67	42.2728	−60	−15	33	63.680	C<B<A
PoCG_R	308	56.985	39	−30	60	45.002	C<B<A
DCG_R	6	41.0275	6	−30	33	14.307	C<B<A
PCUN_L	75	43.3902	−15	−45	66	6.255	C<B<A
SPG_R	29	38.0219	18	−51	63	63.518	A<B<C

ANOVA and Bonferroni post hoc test with FWE correction, voxel-level *p* < 0.001, cluster-level *p* < 0.05. In the post-hoc comparison, A represents the NC group, B represents mild VCI group, C represents the major VCI group. IOG, inferior occipital gyrus; PCG, posterior cingulate gyrus; THA, thalamus; CUN, Cuneus; PoCG, Postcentral gyrus; DCG, median cingulate and paracingulate gyri; PCUN, Precuneus; SPG, superior parietal gyrus; L, left; R, right.

### Differences in functional connectivity

**Figure 2:**
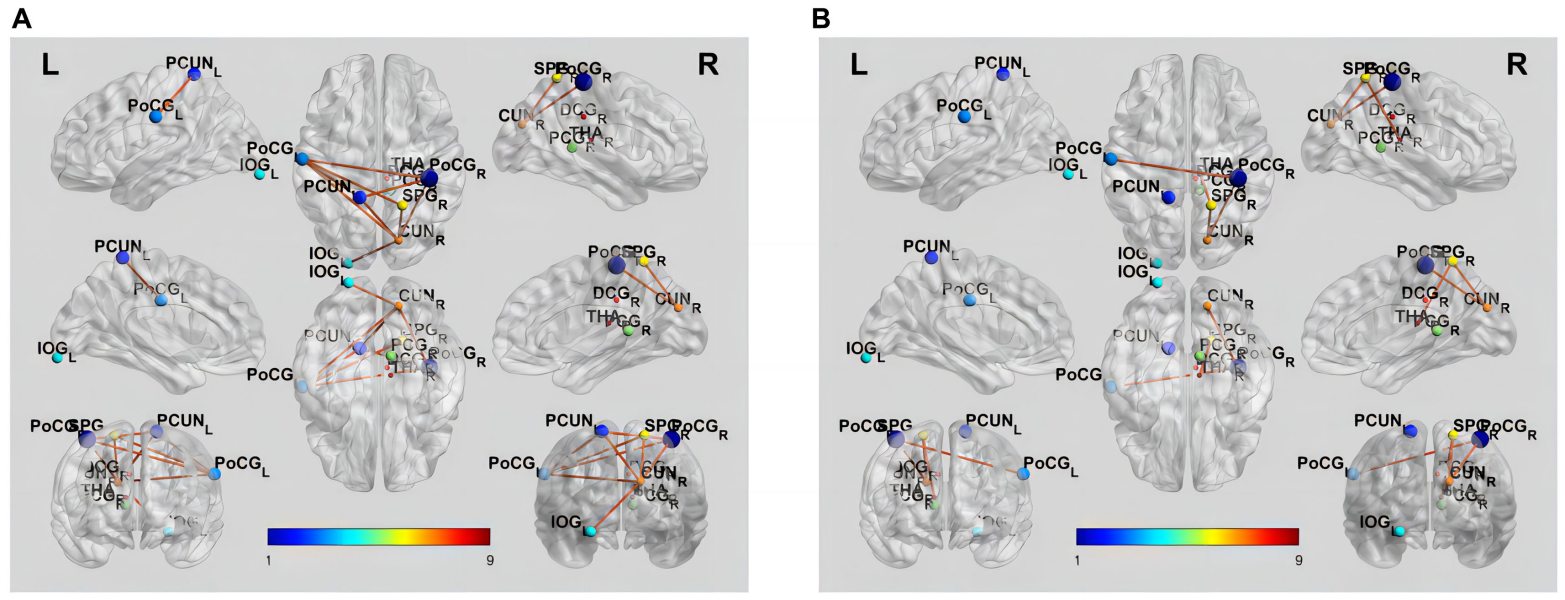
Brain functional connectivity alterations in patients with different groups. **(A)** Increased functional connectivity in NC compared to mild VCI (*p* < 0.05, NBS corrected). **(B)** Increased functional connectivity in mild VCI compared to major VCI (*p* < 0.05, NBS corrected). The brain network edges were extracted from the correlation matrix of rs-fMRI connectivity across the regions of interest. Ball locations represent the peak Montreal Neurological Institute Coordinate System coordinate of differences within clusters, and the size indicates the cluster size. The color of the connections represents differences between groups.

NBS analysis revealed significant differences in FC matrices between mild VCI and NC groups. The subnetwork was comprised of six nodes and nine edges, representing positive functional correlations among NC nodes (Table 3; Figure 2A). Compared to NC group, mild VCI group exhibited reduced positive FC, mainly comprised of nodes in PCUN_L, CUN_R, PoCG_L, PoCG_R, SPG_R and IOG_L.

**Table 3:** Network-based statistics analysis between different brain regions in three groups.

	Connection 1	Connection 2	T-stat
NC-mild VCI	CUN_R	PoCG_R	5.52
	PCUN_L	CUN_R	5.29
	CUN_R	SPG_R	4.45
	CUN_R	PoCG_L	4.17
	PCUN_L	PoCG_L	4.05
	PCUN_L	PoCG_R	3.94
	PoCG_R	PoCG_L	3.69
	PoCG_L	SPG_R	3.64
	CUN_R	IOG_L	3.45
Mild VCI – major VCI	CUN_R	PoCG_R	3.55
	CUN_R	SPG_R	3.50
	PoCG_R	PoCG_L	3.43
	SPG_R	THA_R	3.22

IOG, inferior occipital gyrus; PCG, posterior cingulate gyrus; THA, thalamus; CUN, Cuneus; PoCG, Postcentral gyrus; DCG, median cingulate and paracingulate gyri; PCUN, Precuneus; SPG, superior parietal gyrus; L, left; R, right.

Results also showed a significant difference in the FC matrix between mild VCI and major VCI groups. The subnetwork was mainly comprised of five nodes and four edges, representing positive functional correlations among mild VCI nodes (Table 3; Figure 2B). Compared to mild VCI group, major VCI group exhibited further reduced positive FCs, mainly comprised of nodes in CUN_R, PoCG_L, PoCG_R, SPG_R, and THA_R.

### The mediating effect of abnormal brain activity on cognitive function

**Figure 3:**
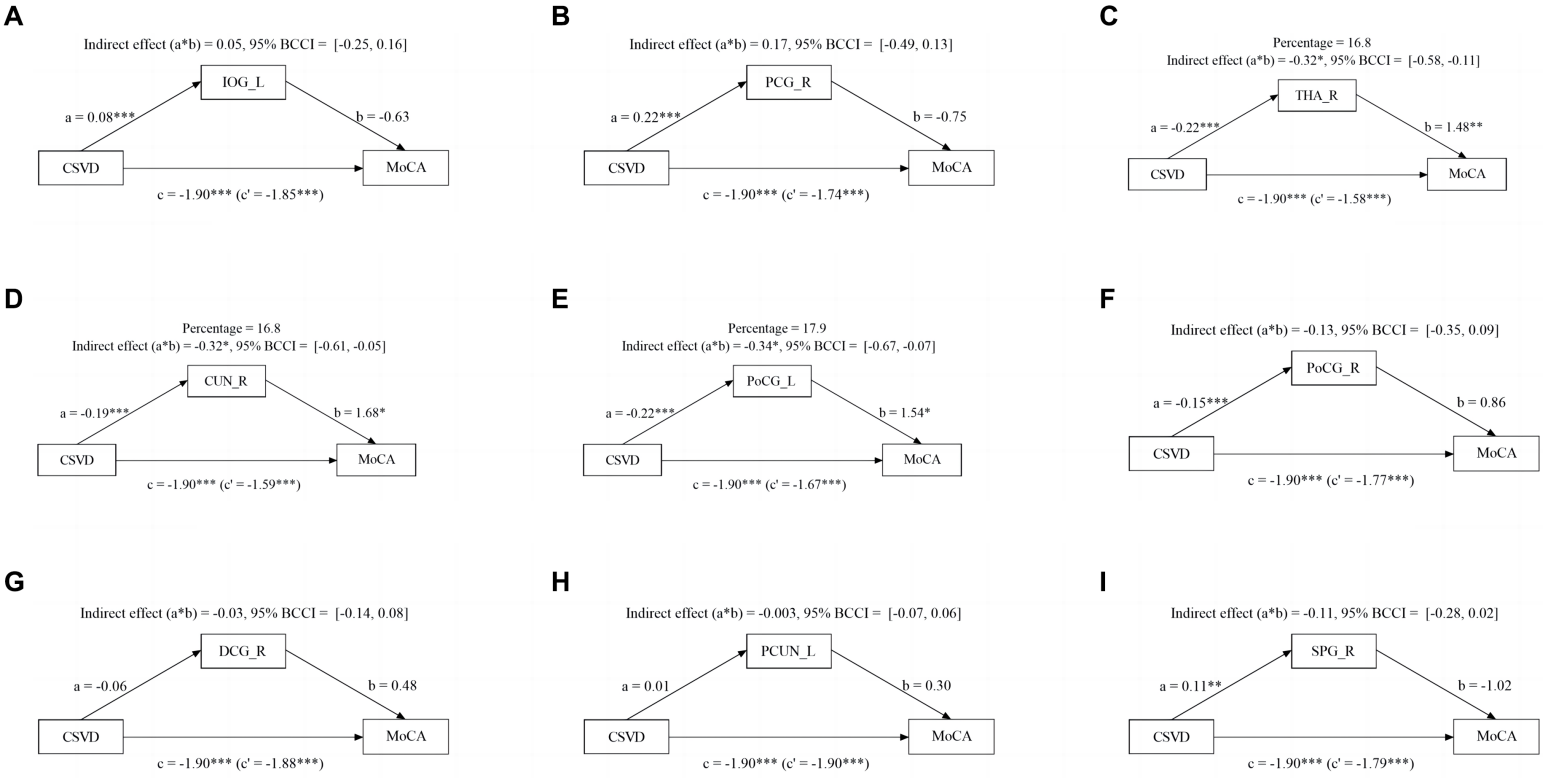
Mediation effect of aberrant brain activity between NC and mild VCI. The zfALFF value of THA_R, CUN_R, and PoCG_L partially mediate associations between CSVD and VCI in NC and mild VCI groups.

**Figure 4:**
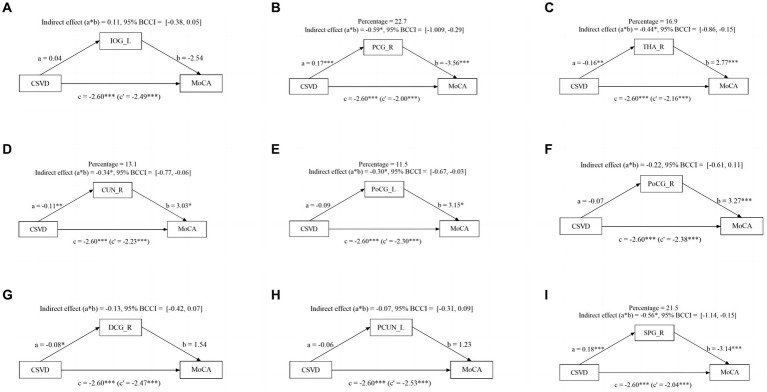
Mediation effect of aberrant brain activity between mild VCI and major VCI. The zfALFF value of PCG_R, THA_R, CUN_R, PoCG_L, and SPG_R partially mediate associations between CSVD and VCI in mild VCI and major VCI group.

Given the significant influence of CSVD and abnormal brain activity on cognitive function, we conducted a mediation analysis to explore the role of abnormal brain activity in mediating cognitive impairment. The mediation analysis depicted in Figure 3 revealed a noteworthy indirect impact of THA_R, CUN_R, and PoCG_L on MoCA scores via VCI progression (from NC to mild VCI), attributing 16.8, 16.8, and 17.9% to the mediation effect, respectively. Similarly, in Figure 4, mediation analysis unveiled a substantial mediating effect of PCG_R, THA_R, CUN_R, PoCG_L, and SPG_R on MoCA scores through VCI progression (from mild to major VCI), with 22.7, 16.9, 13.1, 11.5, and 21.5% mediating effects, respectively. However, no other brain regions demonstrated a significant partial mediation in the association between CSVD and cognitive impairment.

## Discussion

In this study, we aim to identify the changes in aberrant brain activity and network connectivity between mild and major VCI caused by CSVD. Our study has highlighted the following results: (1) Compared to NCs, VCI patients experience various degrees of changes in baseline brain activity. Specifically, decreased fALFF values were found in THA_R, CUN_R, PoCG_L, PoCG_R, DCG_R, and PCUN_L; while increased activity values were observed in IOG_L, PCG_R, and SPG_R. (2) The changes in FC between FC_CUN_R-PoCG_R_, FC_CUN_R-SPG_R_, and FC_PoCG_L-PoCG_R_ may underlie alterations of brain networks in VCI patients. (3) The aberrant baseline brain activity and disrupted brain network intensified with cognitive function weaken. The nodal connectivity efficiency of thalamus was involved when cognitive function progressively weakens. (4) We also verified that the increased fALFF values in brain regions THA_R, CUN_R, and PoCG_L may mediate the impact of CSVD total burden on cognitive impairment.

On the clinical setting, these overall findings indicated that rs-fMRI can provide information on cognitive impairments related to CSVD from various perspectives. Studying the mechanisms of CSVD from the perspective of resting-state activity contributes to exploring the neural basis of cognitive impairments in CSVD patients. The altered functional connectivity derived from these abnormal brain activities may play a crucial role in cognitive impairments in CSVD patients.

### Aberrant brain activity in VCI patients

CSVD is primarily associated with small vessel damage in the brain caused by endothelial damage, blood–brain barrier disruption, atherosclerosis, vascular wall hyalinosis, and amyloid deposition, resulting in alterations in the adjacent parenchyma, eventuating in impairment of cognitive and emotional functions (17). Low-frequency oscillations in the BOLD signal reflects cerebral vascular function and can detect cognitive impairments caused by endothelial dysfunction in CSVD patients. A recent study has shown that the ALFF value reflects the stability of spontaneous brain activity and can detect changes in intrinsic brain activity in CSVD patients with cognitive impairment, which is crucial for early treatment and alleviating the progression of CSVD disease (18). Another study indicated a reasonably consistent regional variation in the magnitude of low frequency oscillations in relation to the severity of persistent markers of CSVD, suggesting its potential as a novel index for evaluating endothelial dysfunction of CSVD (19). Consistent with these findings, our study revealed widely altered fALFF in VCI subjects, mainly manifested as decreased THA_R, CUN_R, DCG_R, PCUN_L, PoCG_L, and PoCG_R. Ni et al. suggested that the decrease of ALFF in individuals with LI, might result from compensatory vascular damage (20). Another study reported that cognitive impairment associated with WMH was connected with an augmented number of regions exhibiting abnormal ALFF values (21). A study found that abnormal brain activity were prominent among the DMN, frontoparietal network, sensorimotor network, and somatosensory network in CMB patients (22). In this study, we found an increase in fALFF in the parietal and occipital lobes, including IOG_1, PCG_2, and SPG_2, which may indicate that compensatory mechanisms and cognitive reserves play an important role in this process. The occipital lobe is involved in visual memory processing and stores visual information in working memory through interaction with other brain regions (23). Meanwhile, the parietal cortex encodes both the perceived image and transformed content (24). They both highlight the importance of the interaction between the parietal and occipital lobes in visual memory processing (25). Thus, the abnormal activity in the parietal-occipital brain regions is closely associated with cognitive processes such as processing speed.

### Disrupted functional connectivity in VCI patients

The brain activity abnormalities associated with CSVD are diverse and complex. Vipin et al. found cerebrovascular disease patients had more early and severe structural connectivity disruptions than control groups, providing important evidence for the impact of cerebrovascular disease on early structural network disruptions (26). Cai et al. reported that the alterations in the brain network’s structure due to interactions within the resting-state network are closely linked to the pathophysiology of cognitive impairment (27). Liu et al. found that both CSVD patients with cognitive impairment and those without cognitive impairment have varying degrees of reduced network connectivity (28). Consistent with the above study findings, our study revealed widely altered static FC in CSVD subjects especially in those with VCI. Our study discovered functional network anomalies concentrated in the brain regions of PCUN_L, CUN_R, PoCG_L, and PoCG_R, these anomalies extended into peripheral areas, as presented. The functional connectivity of PCUN_L, a crucial component of the DMN, experienced impairment, leading to damage within the DMN, it is consistent with previous research (29). However, a study found that FC is not affected by CSVD clinical features such as imaging markers (30). This is different from our results, the variation could stem from significant CSVD risk factors, like age and hypertension, among the subjects. In their study, the brain’s FC abnormalities induced by CSVD might be overshadowed by other factors, potentially undermining the reliability of the results.

### Progressive aberrations in brain activity and disruptions in network connectivity are associated with a decline in cognitive function

Our results revealed that individuals with major VCI exhibit more severe aberrant activity and disruption network connectivity in specific brain regions compared to mild VCI. Firstly, we found that as cognitive impairment weakens, the abnormality of fALFF gradually increases. Diciotti et al. reported that the baseline brain activity were significantly negatively correlated with MoCA scores, which indicated more severe cognitive impairment linking to higher regional abnormal brain activity (31). Ni et al. reported that, in LI-related MCI patients, the MoCA scores showed a relatively weak correlation with ALFF values in specific brain domain (20). Su et al. reported that the ALFF z-scores for the bilateral superior frontal gyrus were negatively associated with cognitive performance, whereas those of the right precuneus and cuneus were positively correlated with the neuropsychological test score (32). Song et al. concluded that there is a wide range of dynamic abnormalities of spontaneous brain activity in patients with CSVD, in which the abnormalities of this activity in specific brain regions are related to cognitive impairment degree (33). Secondly, compared with the mild VCI group, the FCs of the major VCI group were further reduced in several nodes and edges. Sang et al. reported that more reductions were found in nodal efficiency in the prefrontal and temporal cortices for major VCI than for mild VCI (13). In another study about the alteration of dynamic functional connectivity patterns in CSVD, the authors found that major VCI had similar but more extensive changes in the temporal properties of brain states compared to mild VCI. Furthermore, switching from weak connectivity state to strong connectivity state was more difficult in patients with major VCI than in patients with mild VCI. The results revealed that not only the transition to but also maintenance in strongly connected states became increasingly difficult when CSVD-related cognitive impairment progressed into a more severe stage (34). In addition, the Instrumental Activities of Daily Living scores, which was used to differentiate the severity of VCI, were also associated with FC in specific brain regions (35). These findings suggest that brain network connectivity is progressively disrupted as cognitive impairment caused by CSVD gradually increases. Finally, the nodal connectivity efficiency of thalamus was affected when cognitive function progressively weakens. In this study, the FC decline mainly involved in the thalamus in mild VCI compared to major VCI, which plays an essential role in both the salience network and the DMN. The attenuation of thalamic FC is an important neural basis for cognitive decline in patients with CSVD (36). FC disruption in the thalamus may be a potential mechanism for the occurrence and development of cognitive impairment in CSVD (37). Li et al. advocated that the damage to specific white matter fibers projection from the thalamus to the cortex is a imaging marker of CSVD patients with deteriorated cognitive impairments (38).

### Mediation effect of aberrant brain activity in cognitive impairment

We conducted mediation analysis to investigate the potential link between CSVD burden, fALFF index, and cognitive function. Our findings indicate that THA_R, CUN_R and PoCG_L may partially mediate the relationship between CSVD burden and MoCA scores in NC and mild VCI patients. Additionally, PCG_R, THA_R, CUN_R, PoCG_L, and SPG_R partly mediate this relationship in mild VCI and major VCI patients, suggesting different pathways to cognitive impairment caused by CSVD burden. Abnormal activity in specific brain regions partly explains the connection between increased CSVD burden and cognitive impairment. Prior studies have demonstrated that cerebral vascular disease interferes with the structural brain network of patients with small vessel disease (39). The different pathological processes associated with CSVD imaging markers may directly or indirectly impact white matter tract integrity (40). This impact may, in turn, cause gradual reductions in functional connectivity, resulting in disconnection between cortical regions and consequent cognitive impairment, which confirm our research conclusion.

### Limitations

Several limitations should be addressed in this study. Firstly, this is a single-center study, and this should be validated in future research. Secondly, although this study found that CSVD has an impact on the cognitive impairment in different brain areas, after all, it is only a cross-sectional study, and no causal inferences or directionality could be made. Thirdly, the statistical power of our study findings is limited due to its relatively small sample size. Lastly, the current study exclusively explored the differences between brain functional networks in intergroup settings. Further research is required to investigate the differences and connections in the topological properties between brain functional networks.

## Conclusion

In summary, the abnormal brain activities observed in regions such as THA_R, CUN_R, and PoCG_L, along with their corresponding abnormal functional connections, are pivotal in VCI individuals. These aberrant brain activities and disrupted functional connections serve as the neural basis underpinning cognitive impairment in CSVD. Our study indicates that as cognitive impairment due to VCI progresses, there is a concomitant escalation in abnormal brain activity and network connectivity disruptions. Furthermore, the disruption in functional connectivity in the thalamus may represent a potential mechanism contributing to cognitive decline in VCI patients.

## Data availability statement

The raw data supporting the conclusions of this article will be made available by the authors, without undue reservation.

## Ethics statement

The studies involving humans were approved by the Ethics Committee of the Nanxishan Hospital of Guangxi Zhuang Autonomous Region. The studies were conducted in accordance with the local legislation and institutional requirements. The participants provided their written informed consent to participate in this study.

## Author contributions

RM: Writing – original draft, Writing – review & editing, Formal analysis, Conceptualization, Supervision, Methodology, Data curation. PY: Writing – original draft, Writing – review & editing, Formal analysis, Conceptualization, Methodology, Data curation. XQ: Writing – original draft, Writing – review & editing, Funding acquisition, Formal analysis, Methodology, Data curation. WZ: Writing – review & editing, Data curation. XL: Writing – original draft, Writing – review & editing. BH: Writing – review & editing, Data curation. XZ: Writing – original draft, Writing – review & editing, Funding acquisition, Conceptualization, Supervision, Methodology, Project administration.

## Footnotes

^1^Available at: https://restfmri.net

^2^ https://sites.google.com/site/bctnet/comparison/nbs

^3^ https://www.nitrc.org/projects/bnv/

## Glossary

CSVD: Cerebral small vessel disease
MRI: Magnetic resonance imaging
rs-fMRI: Resting state functional MRI
BOLD: Blood oxygenation level dependent
VCI: Vascular cognitive impairment
FOV: Field of view
fALFF: Fractional amplitude of low-frequency fluctuation
FC: Functional connectivity
NC: Normal control
SD: Standard deviation
MOCA: Montreal cognitive assessment
IOG: Inferior occipital gyrus
PCG: Posterior cingulate gyrus
THA: Thalamus
CUN: Cuneus
PoCG: Postcentral gyrus
DCG: Median cingulate and paracingulate gyri
PCUN: Precuneus
SPG: Superior parietal gyrus
L: Left
R: Right
